# Emergence of Daptomycin Resistance in Daptomycin-Naïve Rabbits with Methicillin-Resistant *Staphylococcus aureus* Prosthetic Joint Infection Is Associated with Resistance to Host Defense Cationic Peptides and *mprF* Polymorphisms

**DOI:** 10.1371/journal.pone.0071151

**Published:** 2013-08-19

**Authors:** Nagendra N. Mishra, Soo-Jin Yang, Liang Chen, Claudette Muller, Azzam Saleh-Mghir, Sebastian Kuhn, Andreas Peschel, Michael R. Yeaman, Cynthia C. Nast, Barry N. Kreiswirth, Anne-Claude Crémieux, Arnold S. Bayer

**Affiliations:** 1 Division of Infectious Diseases, Los Angeles Biomedical Research Institute at Harbor-University of California at Los Angeles Medical Center, Torrance, California, United States of America; 2 David Geffen School of Medicine at University of California at Los Angeles, Los Angeles, California, United State of America; 3 Public Health Research Institute Tuberculosis Center, Newark, New Jersey, United States of America; 4 Bichat-Claude Bernard Hospital, Paris, France; 5 EA 3647 Versailles St-Quentin Univ., R-Poincaré Hospital, Garches, France; 6 Interfaculty Institute of Microbiology and Infection Medicine, University of Tuebingen, Tuebingen, Germany; 7 Division of Molecular Medicine, Harbor- University of California at Los Angeles Medical Center, Torrance, California, United States of America; 8 Cedars-Sinai Medical Center, Los Angeles, California, United States of America; University Hospital Münster, Germany

## Abstract

**Background:**

Previous studies of both clinically-derived and *in vitro* passage-derived daptomycin–resistant (DAP-R) *Staphylococcus aureus* strains demonstrated the coincident emergence of increased DAP MICs and resistance to host defense cationic peptides (HDP-R).

**Methods:**

In the present investigation, we studied a parental DAP-susceptible (DAP-S) methicillin-resistant *Staphylococcus aureus* (MRSA) strain and three isogenic variants with increased DAP MICs which were isolated from both DAP-treated and DAP-untreated rabbits with prosthetic joint infections. These strains were compared for: *in vitro* susceptibility to distinct HDPs differing in size, structure, and origin; i.e.; thrombin-induced platelet microbicidal proteins [tPMPs] and human neutrophil peptide-1 [hNP-1]; cell membrane (CM) phospholipid and fatty acid content; CM order; envelope surface charge; cell wall thickness; and *mprF* single nucleotide polymorphisms (SNPs) and expression profiles.

**Results:**

In comparison with the parental strain, both DAP-exposed and DAP-naive strains exhibited: (**i**) significantly reduced susceptibility to each HDP (*P*<0.05); (**ii**) thicker cell walls (*P*<0.05); (**iii**) increased synthesis of CM lysyl-phosphatidylglycerol (L-PG); (**iv**) reduced content of CM phosphatidylglycerol (PG); and (**v**) SNPs within the *mprF* locus No significant differences were observed between parental or variant strains in outer CM content of L-PG, CM fluidity, CM fatty acid contents, surface charge, *mprF* expression profiles or MprF protein content. An isolate which underwent identical *in vivo* passage, but without evolving increased DAP MICs, retained parental phenotypes and genotype.

**Conclusions:**

These results suggest: **i**) DAP MIC increases may occur in the absence of DAP exposures *in vivo* and may be triggered by organism exposure to endogenous HDPs: and ii) gain-in-function SNPs in *mprF* may contribute to such HDP-DAP cross-resistance phenotypes, although the mechanism of this relationship remains to be defined.

## Introduction

*S. aureus* is a prominent human pathogen which can cause severe infections including endocarditis, septicemia, and osteomyelitis [1]–[3]. DAP is a lipopeptide antibiotic with potent activity against Gram-positive bacteria, including multidrug-resistant *S. aureus*. Evolution of DAP-R in *Staphylococcus aureus* during therapy is a growing concern, especially in patients with bone and joint or endovascular infections, when treated by DAP or vancomycin [4]. The emergence of DAP-R strains was also recently observed in a rabbit MRSA prothetic joint infection model [5], which closely parallels similar post-operative infections in human [6]. DAP-R strains emerged in 6/10, and 3/12 rabbits treated with DAP or vancomycin monotherapy, respectively [5], [7]. Interestingly, spontaneous emergence of MRSA with decreased susceptibility to DAP was detected in 2/9 untreated control animals with joint infection. This observation suggested that endogenous host factors interacting with the microorganism during the course of infection could promote the DAP-R phenotype.

We have previously documented *in vitro* “cross-resistance” between DAP and cationic host defense peptides (HDPs) from neutrophils and platelets in *S. aureus* isolates obtained from patients failing DAP therapy [8], [9]. This same cross-resistance phenomenon has been seen in MRSA strains developing DAP-R following serial *in vitro* passage in this agent [10]. This concept was supported by the observation that among 47 DAP-susceptible (S) MRSA bloodstream isolates from DAP-naïve patients, higher DAP MICs (although still in the susceptible range) tracked with reduced susceptibility *in vitro* to sublethal concentrations of platelet-derived, but not PMN-derived HDPs [11]. The mechanism(s) involved in the emergence of such DAP-HDP cross-resistance are not well-defined. The above MRSA strain-set isolated from rabbits with prosthetic joint infections in the presence or absence of DAP therapy provided a unique opportunity to study the influence of endogenous exposures of *S. aureus* strains to specific HDPs, with respect to *in vivo* evolution of the DAP-R phenotype.

Moreover, the current study included specific evaluation of potential correlates of *mprF* point mutations with DAP-HDP cross-resistance phenotypes in MRSA strains from the above animal model, focusing on: **i**) alterations in cell membrane (CM) physiology and metabolism (surface charge; fatty acid content; phospholipid profiles and phospholipic asymmetry; and fluidity); **ii**) cell wall thickness; and **iii**) expression profiles, point mutations and synthetic functions of *mprF*. The *mprF* gene product is of particular relevance in this regard, as it is intimately involved in maintaining bacterial cell surface charge, and has been previously implicated in the DAP-R phenotype [4], [8]–[10].

Although the official term for reduced susceptibility to daptomycin is “*daptomycin nonsusceptibility*”, we will employ the term “*daptomycin-resistance*” (DAP-R) for ease of presentation.

This work was presented in part at the 51^st^ Interscience Conference on Antimicrobial Agents and Chemotherapy, Chicago, IL; USA, Sept 17–20, 2011; abstract #C1-1775 [7] .

## Materials and Methods

### Bacterial strains

The bacterial strains used in this study are listed in **Table 1** (including their antibioitic susceptibility profiles, their *mprF* mutations, if present, and their animal isolation descriptions). Four MRSA strains were primarily used in this study, including: **i**) a parental DAP-S strain used to induce prosthetic joint infection in rabbits (L-271); and **ii**) three strains with increased DAP MICs isolated from either DAP-treated or DAP-untreated rabbits (L-8 and L56; and L-16, respectively) [5]. The two strains with increased DAP MICs obtained from DAP-treated animals (L-8 and L-56) were isolated at 17 d post-infection, following 7 d of DAP therapy [5]. The one strain with increased DAP MICs in the absence of DAP therapy was also obtained at sacrifice after 17d of infection. The details of the animal model, including induction of infection, DAP treatment regimens and therapeutic outcomes have been recently detailed [5]. For selected studies (especially in which phenotypic or genotypic metrics differed between parental L-271 vs L-8, L-16 and L-56 strains), an additional strain (L-76) was employed. This isolate, obtained from bone cultures of a DAP-untreated animal with prosthetic joint infection at 30 d post-infection, maintained a near parental-level DAP MIC (0.38 µg/ml).

**Table 1:** Antimicrobial susceptibilities and *mprF* single nucleotide polymorphisms.

Strains	DAP (µg/ml)	VAN (µg/ml)	OX (µg/ml)	*mprF* SNPs	Days of DAP Treatment	Rabbits sacrificed post infection (days)
271 (Parent)	0.125	3	12	-	-	-
L8	2	4	2	L291I	7	17
L16	0.75	3	6	W424A	0	17
L56	2	3	4	P314L	7	17
L76	0.38	3	24	None	-	30

Isogenicity of the above five study isolates was investigated by profiling: **i**) PFGE patterns; **ii**) *agr* types; **iii**) *spa* types; **iv**) SCC*mec* types; **v**) presence of *pvl* and *acme* genes; and **vi**) clonal complex types (inferred from *spa* types). All assays were performed by standard methodologies [12]–[15] .

The minimal inhibitory concentrations (MICs) of the strains to DAP, vancomycin (VAN) and oxacillin (OX) were determined by standard Etest (AB Biodisk, Dalvagen, Sweden) on Mueller–Hinton agar (MHA) plates, following the manufacturer's protocol [Difco Laboratories, Detroit, MI]. DAP-R was defined as an Etest MIC of ≥2 µg/ml [16].

### Population analyses

We performed DAP population analyses of the selected strain-set by standard protocols [8]. Briefly, the study strains were grown up overnight and the cells were washed with the PBS (phosphate buffer saline), adjusted to OD_600 nm_ at 1.00 (∼10^9^ CFU/ml). Then, 10 µl of the bacterial suspension was added on Mueller- Hilton broth agar plates containing DAP, ranging from 0.062 to 16 µg/ml to encompass the range of sublethal-to-lethal drug levels. Colonies counts were quantified after 24 hour incubation at 37°C and the viable count plotted against DAP concentrations.

### Host defense peptides (HDPs)

Thrombin-induced platelet microbicidal proteins (tPMPs) were obtained from thrombin-stimulated rabbit platelets as previously described. This preparation contains several tPMPs, but predominantly tPMP-1 [17]. The bioequivalency (activity in µg/ml) of the tPMP preparation was determined as detailed before, using a *Bacillus subtilis* bioassay [18]. Purified human neutrophil defensin-1 (hNP-1) was purchased from Peptides International (Louisville, KY).

### HDPs susceptibilities

For tPMPs, a microtiter bactericidal assay was carried out in minimal liquid nutrient medium (Eagles minimal essential media [MEM]) in appropriate buffers [9]; the hNP-1 killing assay was performed in 1% BHI +10 mM potassium phosphate buffer (PPB). A final bacterial inoculum of 10^3^ stationary phase CFU was employed. The peptide concentrations used in the 2 h killing assays were: 1.5 or 2.0 µg/ml equivalent for tPMPs; and 5 or 10 µg/ml for hNP-1. After extensive pilot studies, these peptide concentrations were selected based on: (**i**) sub-lethality, with <50% reductions in counts of the parental DAP-susceptible (DAP-S) strain; and (**ii**) encompassing peptide concentrations used in prior investigations of HDP:*S. aureus* interactions [9]. After 2 h peptide exposure, samples were obtained and processed for quantitative culture to evaluate the extent of killing by each HDP condition. Final data were expressed as mean (± SD) per cent surviving CFU/ml. Since there is no *bona fide* “resistance” breakpoint for HDPs, the mean percent survival (± SD) was statistically evaluated for potential correlates of HDP and DAP susceptibility profiles. Data included a minimum of three experiments performed on separate days.

### tPMPs and hNP-1 passage study

For the tPMP passage study, the parental strain (271) was cultured overnight in BHI medium. The initial inoculum of ∼10^5^ CFU/ml was exposed to 0.5 µg/ml equivalence of tPMPs in MEM, and the strain passaged 15 times on successive days. Surviving colonies after each day's passage were stored at −80°C, then used as the starting inoculum for the ensuing passage in tPMP. After 15 d of such passage, the initial inoculum and tPMP levels were both increased (∼10^9^ CFU/ml [OD_600_ = 1.00]; and 1 µg/ml equivalence of tPMPs, respectively) to increase selective pressure for peptide-resistant clones. An additional 15 d serial passage was then carried out. An identical protocol for hNP-1 passage was performed, employing this peptide at 10 µg/ml for the entire passage period.

After the 30d passage periods, the comparative susceptibility profiles for the pre-passage and post-passage isolate for tPMPs, hNP-1 and DAP were performed. For tPMPs and hNP-1, the 2 hr killing assay in MEM was used as detailed above [18]. For DAP *in vitro* susceptibility, the standard E-test MICs were determined as above.

To investigate the stability of any peptide-resistant phenotypes that emerged during the 30 d passage period, the post-passage strain was again passaged, but in antibiotic-free BHI medium; the DAP, tPMPs and hNP-1 susceptibility profiles reassessed.

### CM phospholipids (PLs) and aminophospholipid translocation (asymmetry)

Because of its role in lysyl-phosphotidylglycerol (L-PG) synthesis and translocations, the functionality of the *mprF* locus has a major impact on the relative proportions of the principle PLs contained within the CM of *S. aureus* [8]. To investigate potential correlates between *mprF* polymorphisms and CM features, PLs were extracted from study strains under all test conditions as described [8], [10]. The major CM PLs of *S. aureus* (PG; L-PG and cardiolipin [CL]) were separated by two-dimensional thin-layer chromatography (2-D TLC) using Silica 60 F254 HPTLC plates (Merck). Fluorescamine labeling (a fluorophore which does not penetrate the outer CM), combined with ninhydrin staining localization, was used within the 2-D TLC plate assay to assess the translocation of L-PG between the inner-to-outer CM bilayer [8], [10]. First-dimension chloroform-methanol–25% ammonium hydroxide (65∶25∶6, by volume) in the vertical orientation and second-dimension chloroform∶water∶methanol∶glacial acetic acid∶acetone (45∶4∶8∶9∶16, by volume) in the horizontal orientation were used for the separation of the PLs for further quantitation by phosphate estimation. For quantitative analysis, isolated PLs were digested at 180°C for 3 h with 0.3 ml 70% perchloric acid and quantified spectrophotometrically at OD_660_.

As a validation for the 2D-TLC assay above, we used an adaptaption of our previously described annexin V-Ca^++^ assay which measures binding to phosphatidyl serine accessible on the outer CM [‘flipped’] [19]. This assay has been traditionally utilized in eukaryotic systems to identify apoptotic reactions, due to the ability of this fluorophore to bind to and detect outer CM translocation of phosphotidylserine (a negatively-charged PL species not present on the outer CM leaflet of *S. aureus*). We modified this assay as an indirect measure of the relative content of outer CM-translocated, positively-charged L-PG; i.e., the more positively-charged L-PG that is translocated to the outer CM leaflet, the less negatively-charged PL species are available for annexin V-Ca^++^ binding [19]–[21]. Briefly, *S. aureus* cells were grown overnight in BHI broth. After centrifugation, the cell pellet was washed twice and resuspended in binding buffer to OD_600_ = 0.5 (∼10^8^ CFU/ml). Next, 5 µl of APC annexin V (purchased from BD Biosciences; San Jose, CA) was added to the cells, with gentle vortexing followed by incubated at room temperature for 15 min in the darkness. Fluorescence was then acquired for 10,000 cells by flow cytometry (FACScalibur) and analyzed for surface-bound Annexin-V (excitation and emission wavelengths = 650 nm and 660 nm, respectively). Data are expressed in relative fluorescent units (parental strain set at 100%).

### CM fatty acid composition

Given the impact of fatty acid composition on CM adaptability to stress, the fatty acid profile of the parental vs animal passage strains was determined. Approximately 20 mg of bacterial cells were harvested from late log phase growth preparations, and then saponified, methylated, and fatty acid esters extracted into hexane as described previously [8], [10]. The resulting methyl ester mixtures were separated by an Agilent 5890 dual-tower gas chromatograph. Fatty acids were identified by a microbial identification system (Sherlock 4.5; courtesy of Microbial ID Inc., Newark, DE) [8], [10].

### CM fluidity

CM fluidity was determined by fluorescence polarization spectrofluorometry as detailed previously [8]–[10] using the fluorescent probe 1,6-diphenyl-1,3,5-hexatriene (DPH). An inverse relationship exists between polarization indices and the degree of CM order (i.e., lower polarization indices [PI value] denotes a greater CM fluidity [8]–[10]. To address biological variability inherent to membrane dynamics, these assays were performed a minimum of six times for each strain on separate days.

### DNA isolation and targeted *mprF* sequencing

Genomic DNA was isolated from *S. aureus* using the method of Dyer and Iandolo [22]. PCR amplification of the *mprF* ORF was performed as we have previously described, using the primers, *mprF*-F-bam (5′-CCCGGATCCAATTAGAATTGATGTGAAAAAATG-3′) and *mprF*-R-sph (5′-CCCGCATGCAGCGCTTCAGG CATAACTGT-3′) [23]. DNA sequencing of the *mprF* ORFs was kindly performed at City of Hope, Duarte, CA.

### RNA isolation and qRT-PCR analysis for *mprF* transcription

For RNA isolation, fresh overnight cultures of *S. aureus* strains were used to inoculate NZY broth to an optical density at 600 nm (OD_600_) of 0.1. Cells were harvested during both exponential growth (2.5 h) and stationary phase (12 h). Total RNA was isolated from the cell pellets by using the RNeasy kit (Qiagen, Valencia, CA) and the FASTPREP FP120 instrument (BIO 101, Vista, CA), according to the manufacturer's recommended protocols.

Quantitative real time PCR (q-RT-PCR) assay was carried out as detailed previously [24], [25]. Briefly, 1 µg of DNase-treated RNA was reverse transcribed using the SuperScript III first-strand synthesis kit (Invitrogen) according to the manufacturer's protocols. Quantification of cDNA levels was performed following the instructions of the Power SYBR green master mix kit (Applied Biosystems) on an ABI PRISM 7000 sequence detection system (Applied Biosystems) or on a LightCycler using the Quanti Fast SYBR green real-time (RT)-PCR kit (Qiagen). The *mprF*, *dltA*, and *gyrB* genes were detected using specific primers as described before [24], [25]. The *dltA* gene was included in these analyses as it also contributes to surface charge maintenance in *S. aureus* [25].

### Determination of *mprF* transcript half-lives

To determine mRNA stability, transcript synthesis was arrested by the addition of 200 µg/ml rifampicin (Sigma; St. Louis, MO). Aliquots were removed at 0, 1, 2, 3, and 5 min post-transcriptional arrest and total RNA samples were isolated as described before [26], [27] Quantitative real time PCR (qRT-PCR) assays were carried out as described above in triplicate, with 16S rRNA [28] as an internal control. RNA half-lives were determined by linear regression analysis of percent RNA remaining versus time.

### Whole genome sequencing

As a complement to the targeted *mprF* sequencing above, it was also important to obtain a more global comparative genomics profile of the parental vs animal passage strains. Briefly, genomic DNA was extracted using a Wizard genomic DNA purification kit (Promega, Madison, WI), following treatment with 20 µg/ml lysostaphin (Sigma-Aldrich, St. Louis, MO). The Genomic DNA Sample Preparation Kit (Illumina, San Diego, CA) was used to generate paired-end libraries. Fragments of the library were sequenced using Genome Analyzer II (Illumina, San Diego, CA). After trimming the reads for low quality bases, Illumina-sequence reads were mapped against *S. aureus* USA300-FPR3757 genome [29] with the Burrows-Wheeler Alignment Tool (BWA) [30]. The SAM file of BWA outputs was then converted to BAM file using SAMtools [31]. Single nucleotide polymorphisms (SNP) and short ‘indels’ (insertions and/or deletions) were called using the default parameters for SAMtools mpileup utility, followed by bcftools and the vcfutils.pl varFilter script (samtools.sourceforge.net/mpileup.shtml) [31], [32]. SNPs and InDels were annotated by an in-house Perlscript using USA300-FPR3757 genome as the reference [29]. The SNPs and indels were classified as coding region, intergenic region and RNA sequences according to the positions. SNPs in the coding sequences were annotated as synonymous or non-synonymous amino acid substitutions. The sequences reported in this study have been deposited in the National Center for Biotechnology Information (NCBI) Sequence Read and Archive (SRA) database (accession no. SRP025984).

### MprF protein content

To correlate *mprF* gene polymorphisms with quantity of MprF protein, we determined the relative amounts of MprF protein produced by the parental or *in vivo* passage variants in relation to changes in DAP MICs. Flow cytometry employing a translational plasmid-based green fluorescent protein (GFP) reporter system was used. The plasmid employed was pCX-mprF-sfGFP, a derivative of pCX19 [33], containing the gene coding for super-folder (sf) GFP with staphylococcal codon optimization [34] fused in-frame to the 3′ end of *mprF* under control of the xylose-inducible xyl promoter. A 2558-bp DNA fragment including the mprF ribosomal binding site and coding region was amplified from plasmid pRB474-mprF [35] by PCR with primers replacing the stop codon with a PvuI restriction site in a way that allowed an in-frame fusion with sfGFP gene (forward primer: 5′-CATCGAATTATAGGAATAGAGCAAA CAAGC-3′; reverse primer: 5′-GGCCGATCGTTTGTGACGTATTACACGCATTACTTTAG-3′). The resulting plasmid was used to transform *S. aureus* 271, L8, and L16, and sfGFP-mediated fluorescence intensities were compared. In brief, for the flow cytometry assays, *S. aureus* strains containing the plasmid construct expressing the xylose-inducible MprF-GFP fusion protein were grown overnight in Mueller-Hinton broth (MHB) and cultures were diluted to OD_600_ of 0.1 into 5 ml of fresh media which was either xylose-free or containing 0.25% xylose (wt/vol) and incubated with agitation (200 rpm at 37°C for 24 hr). Fluorescence was quantified for 10,000 cell using excitation and emission parameters of 485 nm and 525±25 nm respectively. Mean channel fluorescence units (± SD) were calculated from three separate analyses performed independently.

### Surface charge

The relative positive surface charge of the staphylococcal envelope has been shown to correlate with susceptibility to killing by a variety of cationic HDPs [8]. Of note, two of our target genes-of-interest queried for expression profiles in this investigation (*mprF; dlt* see below) significantly contribute to surface positive charge maintenance [35], [36]. The cytochrome c binding assay was performed as surrogate measure of the relative net positive surface charge of the strain-set as described previously [36], [37]. Briefly, cells were grown overnight in BHI media, washed with 20 mM MOPS buffer (pH 7.0) and resuspended in the same buffer at OD_578_ = 1.0. Cells were incubated with 0.5 mg/ml cytochrome *c* for 10 minutes and the amount of cytochrome *c* remaining in the supernatant was determined spectrophotometrically at OD_530_ nm. The more unbound cytochrome *c* that was detected in the supernatant, the more positively charged the bacterial surface. Data were converted and expressed as mean (± SD) amount of unbound cytochrome c. At least three independent runs were performed on separate days.

### Cell wall thickness

Resistance *in vitro* to DAP in *S. aureus* is frequently correlated with a thickened cell wall phenotype reminiscent of VISA strains [38]. Therefore, cell wall thickness of study strains were, compared by transmission electron microscopy [TEM]; [ 9,10]. The mean thickness (nm ± SD) of 100 cells was determined for the strain-set at a constant magnification of 190,000× (JEOL, Model# 100CX, Tokyo, Japan) using digital image capture and morphometric measurement (Advanced Microscopy Techniques v54, Danvers, MA).

### Statistical analysis

The two-tailed Student T-test was used for statistical analysis of all quantitative data. *P* values of ≤0.05 were considered ‘significant”.

## Results

### MICs

DAP, VAN and OX MICs are shown in **Table 1**. For the two *in vivo*-derived isolates with increased DAP MICs following DAP therapy (L8 and L56), DAP MICs increased 16-fold as compared to the parental strain (271), reaching the DAP-R ‘breakpoint’ of 2 µg/ml. Of note, the DAP MIC of the post-infection isolate from the animal unexposed to DAP therapy (L16) increased 6-fold as compared to the parental strain, although not reaching the DAP-R breakpoint above. Interestingly, a reduction in OX MICs from 2–6-fold was observed in all three post-infection strains, representing the so-called OX-DAP “see-saw effect” [39]–[41]. All VAN MICs were in the VISA range [42]–[44]. A 30d post-infection, DAP-unexposed control isolate (L76) maintained parental-equivalent MICs to all study antibiotics.

### Genotyping

Comparative genotyping confirmed that the parental strain (L271), the three *in vivo* animal passage variant strains with increased DAP MICs (L8, L16, L56), as well as the control animal passage isolate (L76) were of identical *spa* type (YHGFMBQBLO; type 1), inferred clonal complex type [10], SCC*mec* type IV and *agr* type I, and were both *pvl*- and *acme*-negative. All isolates were PFGE-identical (data not shown). These data strongly suggested isogenicity among the strain-set.

### Population analyses

**Figure 1:**
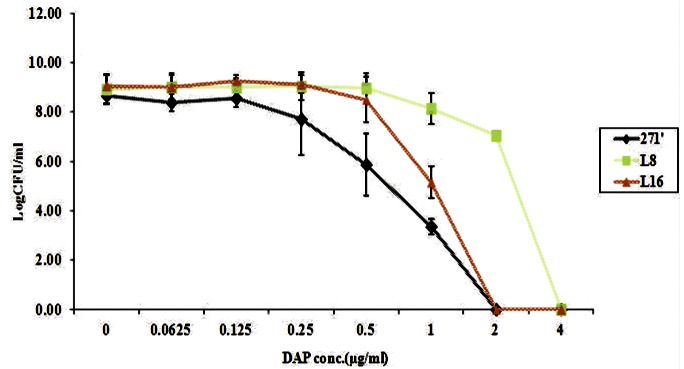
DAP population analysis of the parental vs two animal passage strains.

As noted in **Figure 1**, the DAP population curves of the two variant strains with increased DAP MICs paralleled these strains' differences in MICs. Thus, for both the L8 and L16 isolates, there was a notable shift of the population analysis curves to the right, with the L8 strain curve being substantially more shifted than that for the L16 strain. Of note, there were no DAP hetero-resistant subpopulations detected for either the L8 or L16 isolates.

### HDPs susceptibilities

Among the study strains, both DAP-treated and DAP-untreated *in vivo* strains with increased DAP MICs exhibited reduced susceptibility to killing by HDPs as compared to the parental strain (**Table 2**). These differences were most substantive in comparing the two DAP-treated isolates with the parental strain. Of note, the strain that maintained near parental-level DAP MICs post-*in vivo* passage (L76) remained highly susceptible to killing by study HDPs (data not shown). All HDP susceptibility profiles were stable on multiple passages in nutrient media *in vitro* (data not shown).

**Table 2:** *In vitro* susceptibility profiles to host defense peptides (HDPs).

% survival of a 10^3^ inoculum after 2 hr peptide exposures				
Strains	tPMPs (2 µg/ml)	tPMPs (1.5 µg/ml)	hNP-1 (10 µg/ml)	hNP-1 (5 µg/ml)
271 (Parent)	19±19	26±30	8±6	28±13
L8	76±27*	81±19*	38±27*	60±26*
L16	58±13*	51±13	17±4*	42±8
L56	71±16*	72±13*	33±20*	50±13*

* ***P*** **-value < 0.05 vs parental strain.**

### HDP passage and stability studies

After 30d passage of the parental DAP-S isolate (271) in sublethal concentration of tPMPs, the DAP MIC had increased substantially into the DAP-R range (2 µg/ml) (**Table 3**). Of interest, passage in sublethal hNP-1 did not induce a similar increase in DAP MICs. Further, as shown in **Table 3**, passage in sublethal tPMPs resulted in significant increases in survivability of post-passage isolates to killing by both tPMPs and higher-concentration (but not lower-concentration) hNP-1. Importantly, after serial passage in drug-free media for 5 consecutive days, both the tPMP-passage and hNP-1 passage isolates had reverted to the parental phenotypes in terms of their DAP MICs and HDP survival profiles (data not shown), indicating that these post-HDP passage phenotypes were unstable.

**Table 3:** *In vitro* susceptibilities of parental strain 271 following 30 d tPMP-passage to tPMPs, hNP-1 and DAP.

Mean % survival (± SD) after 2-h exposure to:							
Strains	tPMP 1.5 µg/ml	tPMP 2 µg/ml	hNP-1 5 µg/ml	hNP-1 10 µg/ml	hNP-1 40 µg/ml	hNP-1 80 µg/ml	DAP MIC µg/ml
271 (Parent)	21±12	19±14	71±7	80±9	68±15	17±1	0.5
Post-tPMP Passage	46±13*	49±14*	95±12	95±5	86±9	58±10*	2
Post-passage in tPMP- free medium	29±9	19±14	100±20	84±15	ND	ND	0.5

* ***P*** **<0.05 vs 271 parental strain pre-passage data; ND – not determined.**

### CM PL composition and aminophospholipid asymmetry

Negatively-charged PG was the predominant CM PL in all four principle study strains (**Table 4**). The proportions of the negatively-charged species, CL, were low, and similar among the strains. The proportions of PG were significantly lower in both DAP-treated and DAP-untreated *in vivo* passaged strains exhibiting increased DAP MICs, as compared to the parental strain (*P*<0.00001). Interestingly, the reduction in PG proportionality was related to a relative increase in amounts of the positively-charged species, L-PG, in these latter isolates. Thus, total L-PG levels were more than 2-fold higher in these DAP-treated and DAP-untreated strains as compared to the parental strain (*P*<0.000001), suggesting adaptive ‘gains-in-function’ via the *mprF* locus (i.e., enhanced L-PG synthesis; [8]–[10], [35]). Of note, the proportion of L-PG that was accessible on the outer CM leaflet was approximately 4–7% of total L-PG in all four strains, suggesting similar levels of MprF translocase (flippase) activity among the strains (**Table 4**). This theme is supported by the annexinV flow cytometry analyses, in which annexinV-Ca^++^ binding was similar in all four study strains (% relative fluorescent units ranging from 57.25±0.47 to 63.59±3.24). Importantly, the L-PG profile (i.e., overall proportionality; translocation; etc) of the L76 control *in vivo* passaged strain was similar to that of the parental strain.

**Table 4:** Cell membrane (CM) phospholipid and asymmetry profiles.

% of total CM phospholipid composition (mean ± SD)					
Strains	Inner CM L-PG	Outer CM L-PG	Total L-PG	PG	CL
L271	13.28±0.30	1.07±0.28	14.35±0.02	83.05±0.77	2.59±0.79
L8	30.86±0.30*	0.89±0.24	31.75±0.54*	66.78±0.36**	1.47±0.17
L16	29.55±1.14*	1.46±0.68	31.01±1.82*	66.12±0.57**	2.87±2.39
L56	36.27±0.56*	2.88±1.24	39.15±0.68*	58.60±1.41**	2.26±0.73
L76	14.76±0.30	2.05±0.80	16.81±0.55	72.27±4.84	11±3.76

* ***P*** **-value<0.000001 vs parental strain;**

** ***P*** **-value<0.00001 vs parental strain.**

### CM fatty acid composition; CM fluidity

The parental strain, as well as the three *in vivo* passaged strains with increased DAP MICs showed a similar CM fatty acid pattern in terms of iso- and anteiso- (branched chain) fatty acids (BCFA), as well as straight-chain saturated and unsaturated fatty acids (SCFA; UFA, respectively) (**data not shown**).

Prior investigations of DAP-R *S. aureus* strains indicated frequent alterations in CM fluidity amongst such isolates [8]–[10]. In the present study, however, no statistically significant fluidity differences were observed among the three animal passage strains with increased DAP MICs as compared to the parental strain (PI values ranging from 0.357±0.04 to 0.377±0.05).

#### *mprF* gene point mutations

All three strains (DAP-treated and DAP-untreated) with increased DAP MICs and reduced HDP killing following *in vivo* passage exhibited non-identical single nucleotide polymorphisms (SNPs) within the *mprF* ORF (**Table 1**). Each of the three SNPs were noted to occur in previously reported “hot spots” within the *mprF* ORF, mostly in the 8^th^–12^th^ transmembrane segments of the protein, which bridges synthase and flipping domains (“central bifunctional domain”) [35]. Of note, the *in vivo*-passaged strain which maintained near parental-level DAP MICs and HDP susceptibility profiles exhibited a parental *mprF* gene sequence. Furthermore, the above-described parental isolate which exhibited unstable increases in its DAP MIC following passage in sublethal tPMPs also retained the parental *mprF* sequence.

### *mprF* and *dltA* expression profiles; surface charge

**Figure 2:**
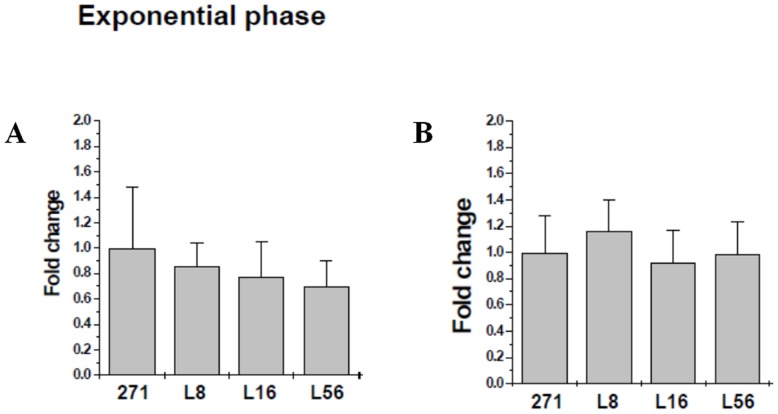
qRT-PCR analyses of *mprF* (A) and *dltA* (B) expression in the study strains. RNA samples were isolated from exponential-phase cultures of the strains and were subjected to qRT-PCR to detect relative transcription levels of *mprF* and *dltA*. Expression levels of *mprF* and *dlt* genes in the parental strain 271 were normalized to 1.

Exponential phase *mprF* and *dltA* expression profiles did not reveal significant differences in comparing the *in vivo*-passaged strains with the parental strain (**Figure 2**). Stationary phase expression of both genes was very low in comparison to exponential phase profiles, and did not indicate any differences in expression amongst the strain-set (data not shown). Paralleling the expression data, all four principle study strains exhibited equivalent levels of relative surface positive charge (>90% repulsion of cytochrome c; data not shown).

### *mprF* mRNA half-lives

All *mprF* mRNA half-lives were <2 min. Thus, analysis of mRNA half-lives revealed no substantive differences among the four study strains in terms of *mprF* transcript stabilities.

### Whole genome sequencing

Whole genome sequencing revealed no significant recombination or excision genomic events (e.g. recombination or excision of sequences) in comparing the three *in vivo* isolates vs. the parental (271) strain. Whole genome sequencing also confirmed the presence of the SNPs identified above within the *mprF* genes in L8, L16, and L56 strains by selected sequence analysis. Of interest, there were relatively few other SNPs identified amongst the *in vivo* passage isolates. Interestingly, several SNPs were identified within *oppB* (oligopeptide ABC transporter permease), *deoC* (deoxyribose-phosphate aldolase), *dut* (dUTP diphosphatase), *chs* (chemotaxis-inhibiting proteins), and *int* (integrase) genes in one or two of the three passage strains (**Table 5**). Of note, all the three passage strains had identical SNPs within two previously uncharacterized genes, SAUSA300_0039 (a hypothetical protein) and SAUSA300_0070 (a putative lysophospholipase). However, no SNPs were found within *rpoB/C*, *yycFG*, *vraRS*, or *cls1* or *cls2* genes in any of three passage strains as compared to the parental strains. These latter loci, along with *mprF* and *dlt* genes, have been variably described to be associated with the DAP-R phenotype in other investigations [7], [9], [10].

**Table 5:** SNPs identified in three animal passage strains vs. the parental 271 strain by whole genome sequencing.

Genes	Description	L8	L16	L56
*oppB* (SAUSA300_0895)	oligopeptide ABC transporter permease	**T234G** **	**-**	**-**
*deoC* (SAUSA300_2090)	deoxyribose-phosphate adolase	**A430C**	**A430C**	**-**
*dut* (SAUSA300_1949)	dUTP diphosphatase	**-**	**T11C**	**T11C; T404A**
*chs* (SAUSA300_1920)	chemotaxis-inhibiting preteins	**T439C**	**A383T; C395A**	**T439C**
*int* (SAUSA300_0799)	integrase	**G296A**	**-**	**-**
SAUSA300_0039	hypothetical protein	**G505A** * **; C507T** * **; C520A** * **; C808T** *	**G505A** * **; C507T** * **; C520A** * **; C808T** *	**G505A** * **; C507T** * **; C520A** * **; C808T** *
SAUSA300_0070	putative lysophospholipase	**G173C** *	**G173C** * **; T175C**	**G173C** * **; T175C**

* **SNPs identified in all three passage strains;**

** **Synonymous substitution.**

### MprF protein content

**Figure 3:**
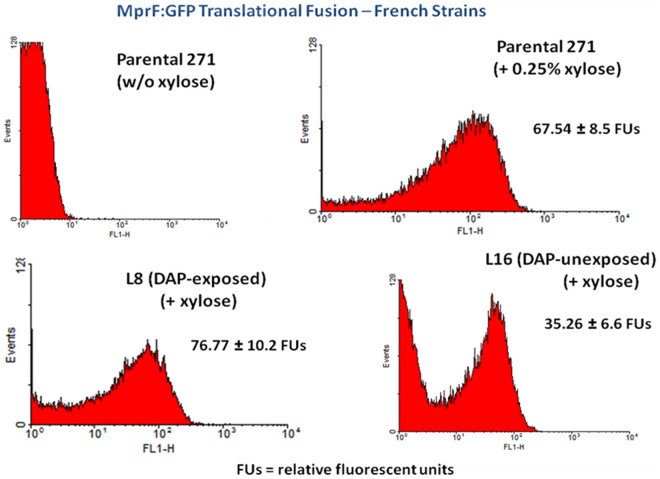
Flow cytometric analyses of the relative amounts of MprF protein content within the parental vs two of the animal passage strains.

Since SNPs found in genes other than *mprF* might have an impact on stability of MprF, we compared fluorescence intensities of MprF-GFP fusion proteins in the parental and two of the mutant strains. Flow cytometric analyses showed that the parental and animal passage strain, L8, had similar levels of MprF produced when protein expression was induced with equal amounts of xylose (**Figure 3**). Of interest, the L16 strain exhibited a substantially lower mean level of MprF protein signal produced as compared to either the parental or L8 isolate. MprF protein was undetectable in control studies performed in the absence of xylose.

### Cell wall thickness

All three isolates obtained following *in vivo* passage exhibited significantly thicker cell walls than the parental strain (20.55±2.24 nm). For example, the cell wall thickness of the DAP-untreated strain, L16, was 21.37±2.67 nm (*P*<0.05 vs parental strain), while those for the two DAP-treated isolates of parental strain 271 (L8 and L-16) were 24.98±2.94 nm and 25.53±2.97, respectively; (both *P*<0.0001 vs the parental strain). The control *in vivo* passage isolate which retained near-parental DAP MICs did not exhibit thickened cell walls as compared to the parental strain (data not shown).

## Discussion

DAP and most cationic HDPs initially target the bacterial CM as a key early part of their lethal mechanisms [8]–[10]. This commonality has led to the notion that “resistance” to killing by one peptide class (e.g., DAP) might be associated with a reduced bactericidal effects among other electrostatically-similar but structurally-unrelated peptide classes (e.g., HDPs). In this regard, it was noted in several studies of individual, clinically-derived, as well as *in vitro* passage-derived DAP-R *S. aureus* strains, that DAP-R tended to track with relative “cross-resistance” to killing by HDPs (HDP-R) [8]–[10]. We recently extended these observations in a more detailed analysis of this DAP-HDP cross-resistance phenomenon in 10 DAP-S/DAP-R isogenic MRSA bloodstream isolate pairs [9]. In this latter investigation, two HDPs, selected for their importance in defense against endovascular infections, were queried [9], including one group from platelets (tPMPs) and one from neutrophils (hNP-1). Of note, DAP-R tracked again with HDP-R in the majority of strains. One major limitation of this prior study was that the DAP-R strains were pre-selected for this resistance phenotype *a priori*. This pre-selection, thus, did not address whether *S. aureus* strains can adapt to reduced HDP and DAP susceptibility phenotypes independently of one another. Moreover, as antibiotic pressure is considered as a leading cause of emergence of antimicrobial resistance, the relative contributions of DAP therapy in relation to endogenous HDP exposures in facilitating development of the ‘cross-resistance’ phenotypes could not be discerned.

Two recent observations from our laboratories provided further insights into the above questions [9], [11]. Among a collection of 47 MRSA bloodstream isolates obtained from patients who had never received DAP (i.e., ‘DAP-naïve’), 12 isolates had relatively high DAP MICs (1 µg/ml), while the remaining 35 isolates had median MICs of 0.25 µg/ml. When comparing the *in vitro* HDP susceptibility profiles of these two MIC groups, increased resistance to killing by tPMPs (but not hNP-1) tracked with the higher DAP MIC group. These data suggested that bloodstream exposure of these isolates (presumably including exposure to platelet-derived HDPs) could “prime for” or “select out” populations of infecting *S. aureus* strains with higher DAP MICs. Prior vancomycin exposures in these patients did not impact the categorization of strains into the lower-versus-higher DAP MIC groups. Moreover, in a recent experimental prosthetic joint infection model, DAP-R MRSA were isolated from both DAP-untreated, as well as DAP-treated animals infected with a DAP-S MRSA parental strain [5]. This study supported the hypothesis that DAP-R could be “naturally” selected *in vivo*, and that this natural selection pressure could be amplified by specific antibiotic treatment. However, it remained to be determined whether the phenotypic and/or genotypic perturbations documented in ‘natural’ DAP-R strains paralled those emerging after selective DAP pressure.

The present investigations were designed to assess concomitant perturbations in HDP and DAP susceptibility phenotypes, as well as relevant genotypes thereof, that arose in the course of a controlled *in vivo* infection, especially focused on events occurring in DAP-naïve scenarios. We took advantage of isolates obtained from the above well-characterized animal model of sub-acute, localized and non-bacteremic staphylococcal infection (prosthetic joint osteomyelitis), with or without DAP exposures [5]. It was reasoned that the major HDPs which the osteomyelitic organisms likely encounter in this prosthetic joint model would be: **i**) neutrophil-derived (related to localized bone-joint abscess formations); and **ii**) to a lesser extent, platelet-derived (related to prosthetic device-induced trauma). These hypotheses provided an important context in which to select specific HDPs for study, as well as in the interpretation of any cross-resistance phenomena.

Several compelling themes emerged from the study data. *First*, three of the aforementioned isolates with increased post-infection or post-therapy DAP MICs were tested for their concomitant susceptibility profiles to the prototypical neutrophil HDP, hNP-1 [8]–[10]. These three isolates were prioritized for investigation based on exhibiting the greatest increases in DAP MICs vs the parental strain (ranging from 6–16-fold). Following *in vivo* passage, all three of these isolates exhibited significantly reduced killing by hNP-1, especially those also exposed to DAP therapy. In contrast, a control *in vivo* passage isolate which maintained near parental-level DAP MICs (L-76) did not exhibit such hNP-1 cross-resistance. The susceptibility profiles of the three strains passaged in animals were also determined against a prototypical platelet tPMP preparation to evaluate potential cross-resistance between hNP-1 and the platelet HDP mixture [8]–[10]. Importantly, even though tPMPs and hNP-1 differ in structure, charge and specific mechanisms of action [45], reduced hNP-1 killing among the animal passage strains tracked closely with reduced tPMP killing of the same isolates. This relationship likely indicates that such peptides share *S. aureus* CM targeting (e.g. initial electrostatic affinity) as a common step in their otherwise distinghishable mechanisms of action. This parallel tracking of hNP-1 and tPMP cross-resistance is reminiscent of several other recent studies in this arena [8]–[10]. These outcomes, along with those from the recent clinical study noted above [11], underscore the concept that endogenous exposures of *S. aureus* to one or more HDPs under sublethal conditions may “prime” such strain populations for selection of either pre-existing or adaptive strains with co-reduced susceptibilities to HDPs and DAP.

*Second*, we attempted to recapitulate the potential mechanism(s) by which the *in vivo* passage isolates adaptively increased DAP MICs in the absence of DAP exposures. Thus, we serially exposed the parental strain to sublethal concentrations of either hNP-1 or tPMPs over a similar 30d time-course, as carried out in the formal animal model studies. Of note, serial passage in a very low and sublethal concentration of tPMPs did induce a substantive increase in DAP MICs, as well as reduced killing profiles for both HDPs above. However, none of these resistance phenotypes was durable following passage in drug-free media, and there were no *mprF* SNPs detected post-HDP passage. Therefore, it seems clear that if *in vivo* exposures to HDPs within a localized infection (as in the current animal model) are playing a role in the emergence of DAP-R, it likely requires exposure of the infecting strain to either: **i**) multiple HDPs from neutrophils and/or platelets; **ii**) combinations of HDPs; **iii**) higher peptide concentrations reflecting those likely to exist *in vivo*; and/or **iv**) additional host factors.

*Third*, the mechanism(s) of *in vitro* adaptive co-resistance to killing by DAP and sublethal levels of HDPs in *S. aureus* remains to be delineated. One prevailing theory in this regard has been the capacity of this organism to modulate its surface charge towards a more relatively positive charge phenotype, potentially creating a “charge-repulsive” surface milieu [8], [35]. However, there were no differences in relative surface charge detected among the parental versus *in vivo*-passage isolates in this investigation, despite the emergence of SNPs within the *mprF* operon during passage *in vivo* [8]. An additional pathway by which *S. aureus* may avoid killing by cationic molecules such as calcium-DAP and HDPs is to alter its relative CM order towards either a much more fluid or more rigid configuration [8]–[10], [46]. This is generally accomplished in Gram-positive bacteria by modifying the relative fatty acid saturation indices and/or proportionality of anteiso-branched chain fatty acids in its CM [46], [47]. However, there were no significant differences in either the CM fluidity index or fatty acid composition among the parental vs animal-passage strains in this study. Other possible mechanisms that have been co-associated with HDP-R and DAP-R include changes in transmembrane potential, carotenoid content, peptide-induced CM permeability, or adaptive responses involving stress response and similar gene pathways. Such mechanisms were not addressed in this investigation.

*Fourth*, all three principle isolates emerging as DAP-R during *in vivo* passage had substantially thicker cell walls than the parental strain. Such thick cell wall phenotypes have been a common, albeit not universal, feature of DAP-R strains [48]. Although controversial, the thickened cell wall phenotype in *S. aureus* has been postulated to be an important contributor to DAP-R either as a mechanical barrier for peptide penetration or via an affinity trapping mechanism [9], [10], [48], [38]. The relative impact of the thickened cell wall upon HDP-induced killing of *S. aureus* has not been elucidated.

*Fifth*, studies from our laboratories and others have suggested that cationic HDPs or DAP may employ negatively-charged lipid domains as putative docking sites during their initial interaction with the target CM [49]. Such events would imply that the proportional CM composition of PG and CL (negatively-charged) vs L-PG (positively-charged) would be expected to influence the amount of cationic peptide eventually binding to the CM. In this respect, it is noteworthy that the relative amount of the negatively-charged PL species, PG, in the *in vivo*-passaged strains with increased DAP MICs was significantly reduced as compared to the parental strain as a reflection of the enhanced synthesis of L-PG [10], [35], [48]. Consistent with this finding, the *mprF* SNPs identified among strains isolated post-*in vivo* passage were localized to the central bifunctional domain of the MprF protein, putatively involved in both L-PG synthesis and/or translocation [35]. These results support an L-PG synthesis ‘gain-in-function’ phenotype among the current study strains with increased DAP MICs. A genetic basis for such a potential gain-in-function remains to be defined, as the observed *mprF* mRNA expression profiles, mRNA half-lives and regulation of MprF protein expression did not differ substantially between the parent and *in vivo*-passaged isolates. Moreover, recent unpublished data document that the relative distribution of the MprF protein within the free CM and septal CM regions are equivalent amongst our study strains (*Kuhn S et al; personal communication*). Collectively, these results suggest that the observed *mprF* mutations seen in the *in vivo* passage strains with increased DAP MICs may affect either MprF structural (e.g., protein conformation) and/or functional characteristics.

*Lastly*, our whole genome sequencing investigations were important in the context of identifying a relatively limited cardre of genes among *in vivo* passage strains within which point mutations emerged. Such data should provide an important framework to further pursue the genetic basis of *in vivo* emergence of the DAP-R phenotype. Of particular interest was the observation of SNPs occurring within the *oppB* gene (oligopeptide ABC transporter permease) in one of the isolates post-*in vivo* passage (L8). The *opp* loci were previously identified in signature-tagged mutagenesis library studies as important virulence genes in multiple animal models, including rabbit endocarditis [50].

We recognize that our current investigations have methodologic challenges which somewhat limit interpretation. For example, the *in vitro* HDP susceptibility testing was performed in rather austere media, in the absence of host factors (e.g., serum or complement proteins, etc.). Moreover, both neutrophils and platelets contain a large cohort of HDPs which were not tested either individually or in combination in this study. Also, the concentrations of HDPs utilized in our *in vitro* assays are undoubtedly lower than bacteria encounter *in vivo*. Moreover, both the host and pathogen are constantly adapting to changing contexts of infection, from the point of initial inoculation to potential long-term persistence, or to host clearance. These, and other factors, alone and in combination are likely critical to selective pressures for immune avoidance placed on *S. aureus* during the course of infection. Further, only a single clonal lineage genotype strain was assessed in the present investigation. It has been well-chronicled that different *S. aureus* clonal lineages are associated with rather distinct clinical outcomes [51]. Finally, there are likely other genes or genetic pathways outside of *mprF* or *dlt* that may be involved in the evolution of increased DAP MICs following *in vivo* passage. However, it was impressive that on comparative whole genome sequencing, only a limited cadre of SNPs were identified among the animal passage strain, primarily in ORFs of unknown functions. It will be pivotal to characterize these loci further through genetic manipulations (e.g. generation of knock-out/overexpression strains) in the context of DAP-R. Moreover, comparative and quantitative gene expression profiles of the parental vs passage isolates may provide additional insights into mechanisms of increasing DAP MICs in the latter strains.

Based on current study, DAP-naïve MRSA strains exposed to HDPs *in vivo* may increase their DAP MICs prior to DAP exposures. In addition, it has also been previousl shown that vancomycin exposures either *in vitro* or *in vivo* can independently lay the foundation for both DAP-R and HDP-R phenotypes (e.g. to tPMPs) [52]. Therefore, we propose the caution against the use of vancomycin in treating DAP-naïve MRSA strains with higher DAP MICs, even if within the “susceptible” range (1–1.5 µg/ml).

In *summary*, *S. aureus* strains isolated from a localized *in vivo* infection model exhibited a cross-resistance phenotype to unrelated HDPs from platelets and PMNs, as well as increased DAP MICs, even in the absence of DAP exposures. These findings are consistent with the concept that encountering specific HDPs during infection may select for surviving strains which are cross-resistant to DAP, and/or “prime” surviving organisms for subsequent and parallel adaptations to both HDPs and DAP. Whether the mechanism(s) of resistance to killing by such diverse cationic peptides among *S. aureus* strains is a single shared pathway or represents multiple distinct pathways remains to be determined.
